# MYH9 confers doxorubicin resistance by stabilizing GRP78 and suppressing the PERK signaling-driven apoptotic pathway in breast cancer

**DOI:** 10.3389/fonc.2026.1923603

**Published:** 2026-10-02

**Authors:** Jing Jiang, Tianzi You, Ziyi Zhao, Binlei Sheng, Zhanqi Sun, Xujun Li

**Affiliations:** 1 Department of Breast Surgery, Ningbo No. 2 Hospital, Wenzhou Medical University, Ningbo, Zhejiang, China; 2 Department if Dermatology, Traditional Chinese Medicine Hospital of Ninghai County, Ningbo, Zhejiang, China; 3 Health Science Center, Ningbo University, Ningbo, Zhejiang, China; 4 School of Medicine, ShaoXing University, ShaoXing, Zhejiang, China; 5 Department of Oncology, Ningbo Second Hospital, Ningbo No. 2 Hospital, Wenzhou Medical University, Ningbo, Zhejiang, China

**Keywords:** ADR-resistant, breast cancer, endoplasmic reticulum stress, GRP78, MYH9

## Abstract

**Background:**

MYH9 is upregulated in various malignancies, but its role in breast cancer chemoresistance remains unclear. We investigated whether MYH9 regulates doxorubicin (ADR) resistance via GRP78 stabilization and PERK pathway modulation.

**Methods:**

MYH9 expression was analyzed in breast cancer tissues and cell lines by immunohistochemistry and western blot. ADR-resistant MCF-7 and MDA-MB-231 cell lines were established. MYH9 knockdown/overexpression and GRP78 overexpression were achieved by lentiviral transduction. Protein interactions, degradation assays, PERK signaling, apoptosis, and *in vivo* drug responses were evaluated.

**Results:**

MYH9 was significantly upregulated in breast cancer tissues, particularly in triple-negative breast cancer (95.2%), and further elevated in ADR-resistant cells. MYH9 overexpression enhanced ADR tolerance, while knockdown sensitized resistant cells *in vitro* and *in vivo*. Mechanistically, MYH9 directly bound GRP78 (but not CALR or GRP94), inhibiting ubiquitin-mediated proteasomal degradation. MYH9 depletion reduced GRP78 levels and activated the PERK pathway (p-eIF2α, ATF4, CHOP), promoting apoptosis via Bax/Bad upregulation and Bcl-2 downregulation. ERS induction with thapsigargin enhanced ADR sensitivity, whereas 4-PBA attenuated it. GRP78 overexpression rescued ADR resistance, confirming GRP78 dependency.

**Conclusion:**

MYH9 confers ADR resistance in breast cancer by stabilizing GRP78 and suppressing PERK-driven apoptosis, identifying the MYH9/GRP78 axis as a potential therapeutic target for overcoming chemoresistance.

## 1 Introduction

Breast cancer is the most prevalent malignancy among women worldwide, and chemotherapy remains a cornerstone of treatment, particularly for advanced disease (1, 2). However, the development of chemoresistance remains a major cause of treatment failure and patient mortality, with approximately 20% to 30% of patients at high risk of recurrence and metastasis following chemotherapy (3–5). Therefore, elucidating the mechanisms underlying chemoresistance is critical for improving therapeutic outcomes.

The myosin superfamily plays pivotal roles in diverse cellular processes, including intracellular transport, cytoplasmic streaming, and cell motility (6–9). Non-muscle myosin heavy chain-9 (MYH9) exerts its biological functions primarily through actin binding and ATP hydrolysis (10). While early studies linked MYH9 mutations to hereditary hematological disorders (11), accumulating evidence has established its involvement in cancer progression (12–15). Notably, MYH9 is implicated in the maintenance of cancer stemness and the regulation of drug resistance across multiple tumor types (16, 17). In breast cancer, MYH9 is upregulated and associated with disease progression (14, 18–20), yet its specific role in chemoresistance remains poorly understood.

The endoplasmic reticulum (ER) is the primary site for protein synthesis, folding, and transport (21, 22). Cellular stressors such as hypoxia, chemical agents, and oxidative stress lead to the accumulation of misfolded proteins in the ER lumen, disrupting ER homeostasis—a condition referred to as endoplasmic reticulum (ER) stress (21–24). In response to ER stress, the unfolded protein response (UPR) is activated as a protective signaling cascade (23, 24). The UPR is mediated by three ER membrane sensors, for example, PERK, ATF6, and IRE1, which are normally held in an inactive state by binding to the ER chaperone glucose-regulated protein 78 (GRP78) (25, 26). Upon stress, misfolded proteins competitively bind to GRP78, releasing the sensors to activate downstream signaling (27, 28). While ATF6 and IRE1 primarily promote adaptive responses by enhancing misfolded protein degradation, the PERK pathway is the principal trigger of the apoptotic signaling cascade (25–28). Thus, GRP78 functions as a central rheostat that determines cell fate under stress by balancing survival and apoptosis.

Increasing evidence links ERS to chemoresistance (25, 26). During chemotherapy, the accumulation of misfolded proteins initially activates PERK-mediated apoptosis (29, 30). However, cancer cells can acquire tolerance by upregulating protective mechanisms, including enhanced GRP78 expression, which suppresses PERK-driven apoptosis and confers drug resistance. For instance, afatinib treatment induces PERK dissociation from GRP78, leading to PERK oligomerization and activation of the PERK-eIF2α-ATF4 apoptotic axis in liver cancer cells (31). Conversely, upregulation of GRP78 by p38 signaling enables dormant tumor cells to resist chemotherapy (30). Although GRP78-mediated ERS signaling is clearly associated with drug resistance, the molecular mechanisms governing GRP78 stabilization during chemoresistance remain unclear.

In this study, we demonstrate that MYH9 is upregulated in breast cancer tissues and cells, and is further elevated in doxorubicin (ADR)-resistant breast cancer cells. Mechanistically, MYH9 directly interacts with GRP78, preventing its ubiquitination and degradation, thereby stabilizing GRP78 and suppressing PERK-driven apoptosis. Consequently, MYH9 knockdown restores ADR sensitivity by activating the PERK apoptotic pathway, while GRP78 overexpression rescues the resistant phenotype. Our findings reveal a novel MYH9/GRP78 axis that regulates chemoresistance in breast cancer through modulation of the ERS apoptotic pathway, providing a potential therapeutic target for overcoming ADR resistance.

## 2 Materials and methods

### 2.1 Cell culture

All cell lines used in this study were cryopreserved and maintained in our laboratory, and the original source is Wuhan Procell Life Science & Technology Co., Ltd. Normal breast epithelial cell line MCF-10A (RRID: CL_0525) were cultured in DMEM/F-12 medium (Invitrogen, USA) supplemented with 5% Donor Equine Serum, 20 ng/mL EGF (Beyotime Biotechnology, Jiangsu, China), 0.5 μg/mL Hydrocortisone (MedChemExpress, USA), 10 μg/mL insulin (Beyotime, China) and 1% MEM Non-Essential Amino Acid Solution (NEAA) (Procell, China) at 37 °C in a humidified incubator with 5% CO_2_. MDA-MB-231 (RRID: CL_0150) and MDA-MB-468 (RRID: CL_0290) cell lines were maintained in DMEM (Procell, China) supplemented with 10% FBS (Procell, China) at 37 °C under a 5% CO_2_-enriched atmosphere. MCF-7 (RRID: CL-0149) cell line was cultured in MEM (Procell, China) supplemented with 10 μg/mL Insulin (Procell, China) and 10% FBS (Procell, China) at 37 °C under a 5% CO_2_-enriched atmosphere. BT-474 (RRID: CL_0040) cell line was cultured in RPMI-1640 (Procell, China) supplemented with 10 μg/mL Insulin (Procell, China), 2 mM L-glutamine (Procell, China) and 20% FBS (Procell, China) at 37 °C under a 5% CO2-enriched atmosphere. HEK293T cells (RRID: CVCL_0063) were maintained in DMEM (Procell, China) supplemented with 10% FBS (Procell, China) at 37 °C under a 5% CO_2_-enriched atmosphere. All cell lines were regularly tested for mycoplasma contamination and confirmed to be mycoplasma-negative.

### 2.2 GEPIA database survival analysis

To evaluate the prognostic significance of MYH9 expression in breast cancer, we performed survival analyses using the Gene Expression Profiling Interactive Analysis (GEPIA) database (http://gepia.cancer-pku.cn/). The GEPIA platform integrates RNA sequencing data from 1070 breast cancer patients from The Cancer Genome Atlas (TCGA) datasets. Patients were stratified into high- and low-MYH9 expression groups based on the median expression level. Kaplan-Meier survival curves for Overall Survival (OS) and Disease-Free Survival (DFS) were generated, and log-rank p-values were calculated to assess the statistical significance of differences between the two groups.

### 2.3 Establishment of ADR-resistant breast cancer cells

To establish the MCF-7/ADR and MDA-MB-231/ADR cell lines, the culture medium contained 20 nM ADR. The cells were continuously exposed to this medium for ten days, after which the ADR concentration was gradually increased over a 6-month period until it reached 1 μM. Thereafter, the cells were maintained continuously in the presence of 1 μM ADR. For the control group, an equal volume of DMSO was added throughout the experiment to prevent the accumulation of stochastic mutations and avoid changes in transcriptional profiles. The drug was removed from the culture medium five days before the cells were used for cellular and molecular biological assays.

### 2.4 MYH9 knockdown and overexpression in breast cancer cells

To generate MYH9-KD cells, breast cancer cells were infected with a lentivirus carrying MYH9 shRNA (5’-GGGTATCAATGTGACCGAT-3’) or NC shRNA (5’-TTCTCCGAACGTGTCACGTTTC-3’). Next, the cells were grown and selected in growth media containing 2 μg/mL puromycin. The puromycin-resistant colonies were then selected, pooled and cultured for further experiments under puromycin-selection conditions.

To generate MYH9-overexpressing cells, breast cancer cells were transduced with a lentiviral vector (PGMLV-CMV-H_MYH9-EF1-mScarlet-T2A-Puro) carrying the full-length MYH9 cDNA, while the empty vector (PGMLV-CMV-H-EF1-mScarlet-T2A-Puro) served as the control. Transduced cells were selected with 2 μg/mL puromycin for two weeks, and stable overexpression was confirmed by western blot analysis.

### 2.5 GRP78 overexpression in breast cancer cells

The human full-length GRP78 cDNA was cloned into the lentiviral expression vector PGMLV-CMV-H_GRP78-EF1-mScarlet-T2A-Puro, generating a GRP78 overexpression plasmid, while the empty vector served as a control. Breast cancer cells were transduced with the respective lentiviral particles, and the culture medium was replaced with fresh medium 24 hours after viral removal. Stable transductants were subsequently selected using puromycin.

### 2.6 Immunohistochemistry

Immunohistochemical analysis was performed to evaluate protein expression in human breast cancer specimens and murine tumor tissues. Briefly, 5 μm-thick tissue sections were mounted on glass slides pre-coated with 3-(triethoxysilyl) propylamine (Merck, Darmstadt, Germany). The slides were then incubated in xylol for one hour and washed through a graded alcohol series. After deparaffinization, antigen retrieval was conducted by microwave heating for five minutes in TEC buffer (0.05 M Tris-HCl, 0.05 M EDTA, 0.02 M sodium citrate, pH 7.8). Endogenous peroxidase activity was subsequently quenched. Sections were incubated with the primary antibody at 4 °C overnight (12 hours), followed by application of a biotin-conjugated rabbit anti-mouse secondary antibody (Vector, Grünberg, Germany) for 30 minutes. Chromogenic development was carried out using diaminobenzidine (DAB) substrate (Sigma, USA) for ten minutes, and nuclei were counterstained with hematoxylin.

### 2.7 Cell viability and colony formation assay

Cell viability and proliferative capacity were evaluated using the MTS [3-(4, 5-dimethylthiazol-2-yl)-5-(3-carboxymethoxyphenyl)-2-(4-sulfophenyl)-2H-tetrazolium, inner salt] assay and colony formation analysis. In the MTS assay, cells were seeded in 96-well plates at a density of 1 × 10^4^ cells per well in 100 µL of culture medium. After adding 20 µL of MTS reagent (Promega, USA), the plates were incubated for 1.5 hours, and the absorbance was measured at 450 nm. All experiments were conducted in triplicate. For the colony formation assay, 500 cells were plated per well in 6-well plates and cultured for 15 days. The resulting colonies were then washed with PBS, fixed with 4% paraformaldehyde for 20 minutes, and stained with 0.1% crystal violet. Colonies were quantified by manual counting under a light microscope.

### 2.8 Endoplasmic reticulum stress inducer and inhibitor treatment

To investigate the role of ERS in ADR resistance, ADR-resistant breast cancer cells were treated with thapsigargin (TG; Sigma-Aldrich, USA, Cat.# T9033), an ER stress inducer, at a concentration of 1 μM for 24 hours, or with 4-phenylbutyric acid (4-PBA; Meilunbio, Dalian, China, Cat.# MC8021), an ER stress inhibitor, at a concentration of 5 mM for 24 hours. Cells treated with an equal volume of DMSO or PBS served as controls.

### 2.9 IC50 determination

To determine the half-maximal inhibitory concentration (IC50) of ADR in wild-type and ADR-resistant breast cancer cells, wild-type MCF-7 and MDA-MB-231 cells, as well as their corresponding ADR-resistant derivatives (MCF-7/ADR and MDA-MB-231/ADR), were seeded in 96-well plates at a density of 5 × 10^3^ cells per well and allowed to adhere overnight. Cells were then treated with serial dilutions of ADR for 48 hours. For ADR-resistant cells (MCF-7/ADR and MDA-MB-231/ADR), the drug concentrations used were 1, 2, 5, 10, 20, 50, 100, and 200 μM. For wild-type cells (MCF-7 and MDA-MB-231), the drug concentrations used were 0, 0.1, 0.5, 1, 2, 5, 10, and 20 μM. Cell viability was assessed using the MTS assay as described above. IC50 values were calculated using GraphPad Prism 9.0 software (GraphPad Software, San Diego, CA, USA) by fitting the dose-response curves with the non-linear regression model (log[inhibitor] vs. response — Variable slope).

### 2.10 Western blot

Proteins were resolved by 12% SDS-PAGE and electrotransferred onto a PVDF membrane. To reduce nonspecific interactions, the membrane was blocked with Tris-buffered saline (TBS) supplemented with 5% (w/v) skim milk for 1 hour at room temperature. Subsequently, the membrane was incubated overnight with the primary antibody. The following day, it was washed with TBST (TBS containing 0.1% Tween 20; four washes, 5 min each) and then probed with a horseradish peroxidase-conjugated secondary antibody (1:10,000 dilution; Bio-Rad) for 1 hour. Following extensive washing with TBST (four washes, 20 min each), protein bands were visualized using enhanced chemiluminescence (ECL).

### 2.11 Caspase 3/7 activity

The Caspase-Glo 3/7 assay kit (Promega, USA) was employed to assess caspase 3/7 activities following the manufacturer’s instructions. Cells were seeded at a density of 1×10^4^ per well in a 96-well plate and subsequently treated with the corresponding processing. After 36 hours, 100 μl of Caspase-Glo 3/7 reagent (Promega, USA) was introduced into each well. The plate was incubated in the dark at room temperature for 30 minutes, after which luminescence was quantified.

### 2.12 Tumorigenicity in nude mice

Nonobese diabetic/severe combined immunodeficiency (NOD/SCID) female mice, approximately five weeks old and weighing ~25 g, were utilized to evaluate *in vitro* oncogenic potential. Cells were washed with PBS, collected at concentration of 5 x 10^5^ cells/mL in physiological saline, and mixed with Matrigel (Becton, Dickinson and Company, USA) at a 2:1 ratio. Mice received a subcutaneous injection of 200 μL (1 x 10^5^ cells) of the cell suspension to induce tumor formation. Seven days later, the mice were intraperitoneally injected with ADR (30 mg/kg) every three days for 4 weeks. After 30 days, mice were euthanized by cervical dislocation, and solid tumors were harvested using surgical scissors.

### 2.13 Breast cancer tissue chip protein expression analysis

A breast cancer tissue microarray was purchased from Zhongke Guanghua (Xi’an) Intelligent Biotechnology Co., Ltd. 145 breast cancer specimens and 30 matched adjacent normal tissues were included in the chip. Of the 145 cancer cases, 30 were classified as stage 1, 70 as stage 2, and 45 as stage 3, with lymph node metastasis present in 49 patients. Patient age at diagnosis was recorded for all cases and categorized into two groups: ≤50 years and >50 years. Hormonal receptor status, including estrogen receptor (ER), progesterone receptor (PR), and human epidermal growth factor receptor 2 (HER2), was recorded for all cases and categorized according to the St. Gallen consensus guidelines. Immunohistochemistry was used to detect the MYH9 expression at each point on tissue microarray.

### 2.14 Assessing GRP78 ubiquitination levels

Cell lysates were generated through incubation of cells in lysis buffer (5 mM piperazine-1,4-bisethanesulfonic acid, 85 mM KCl, and 0.5% Nonidet P-40) for 30 minutes at 4 °C. Subsequently, the lysates were immunoprecipitated with an anti-GRP78 monoclonal antibody (Cell Signaling Technology, USA) for 12 hours at 4 °C, followed by the addition of Protein G–agarose beads (Invitrogen, USA). After a further 3-hour incubation at 4 °C, the complexes were washed three times with ice-cold phosphate-buffered saline (PBS). Immunoblotting was then performed using an anti-ubiquitin antibody (Cell Signaling Technology, USA).

### 2.15 Apoptosis TUNEL staining

To assess apoptosis in mice tumor tissue sections, a TUNEL assay was performed. The sections were first treated with 100 µl of the TUNEL reaction mixture and incubated at 37 °C for one hour. Following three washes with PBS, the samples were labeled with an anti-FITC-alkaline phosphatase conjugat for 30 minutes at 37 °C. After another rinsing step, a substrate solution was applied, and the samples were incubated in the dark for 10 minutes at room temperature. The color reaction was terminated by the addition of ddH_2_O. Finally, the TUNEL-stained specimens were examined using a laser scanning confocal microscope (Leica Microsystems, Buffalo Grove, IL).

### 2.16 Co-immunoprecipitation assays

The interaction between MYH9 and ER chaperones (GRP78, CALR, and GRP94) was examined by Co-IP. In brief, cells were harvested and lysed in ice-cold cell lysis buffer (Beyotime Biotechnology, China) containing 2 mM phenylmethylsulfonyl fluoride (PMSF) protease inhibitor (Solarbio Life Sciences, China). The lysates were clarified by centrifugation and protein concentrations were determined. For the detection of MYH9–GRP78 interaction, lysates were immunoprecipitated with an anti-MYH9 antibody overnight at 4 °C, followed by the addition of Protein G–agarose beads (Invitrogen, USA) and incubation for 4 hours at 4 °C. For the assessment of binding specificity, parallel Co-IP assays were performed using anti-CALR antibody or anti-GRP94 antibody under the same conditions.

After three washes with ice-cold PBS, the Co-IP complexes were collected and subjected to western blot analysis. The MYH9–GRP78 interaction was detected using an anti-GRP78 antibody, while the binding of MYH9 to CALR and GRP94 was assessed by western blotting with an anti-MYH9 antibody.

### 2.17 Statistical analysis

All statistical analyses were performed using SPSS software and GraphPad Prism. Data are presented as the mean ± standard deviation (SD) from at least three independent experiments.

Differences between two groups were analyzed using Student’s t-test (two-tailed). For comparisons involving three or more groups, one-way analysis of variance (ANOVA) was performed. The relationship between MYH9 expression and clinicopathological characteristics was analyzed using the Chi-square test. A P-value < 0.05 was considered to indicate a statistically significant difference.

## 3 Results

### 3.1 MYH9 exhibits high expression in breast cancer and promotes the proliferation of breast cancer cells *in vitro*

To investigate MYH9 expression in breast cancer, we utilized a tissue microarray comprising 145 breast cancer specimens and 30 matched adjacent normal breast tissue. Of the 145 cancer cases, 30 were classified as stage 1, 70 as stage 2, and 45 as stage 3, with lymph node metastasis present in 49 patients. Immunohistochemical staining revealed significantly elevated MYH9 expression in breast cancer tissues compared to normal adjacent tissues (**Figure 1A**; **Table 1**) (χ2 = 16.378, P<0.001). Furthermore, MYH9 expression levels showed a positive correlation with both tumor stage (χ2 = 12.233, P<0.01) and lymph node metastasis status (χ2 = 4.959, P<0.05), but no significant association with patient age at diagnosis (χ2 = 2.325, P = 0.127) (**Table 2**). To evaluate the prognostic significance of MYH9, we performed survival analyses using the GEPIA database. As shown in **Figures 1B**, Kaplan-Meier curves indicated that MYH9 expression was not significantly associated with Overall Survival (OS, Log-rank p=0.94) or Disease-Free Survival (DFS, Log-rank p=0.83) in the total breast cancer cohort. Given the molecular heterogeneity of breast cancer, we further examined MYH9 expression across different subtypes in our clinical cohort. As detailed in **Table 3**, MYH9 expression was significantly upregulated in Triple-Negative Breast Cancer (TNBC) tissues compared to other molecular subtypes (Luminal A, Luminal B, and HER2-enriched), with 95.2% (40/42) of TNBC cases showing high MYH9 expression (P<0.001). In summary, MYH9 is upregulated in breast cancer tissues and shows a distinct association with aggressive phenotypes and the triple-negative subtype, highlighting its potential role in tumor progression.

**Figure 1:**
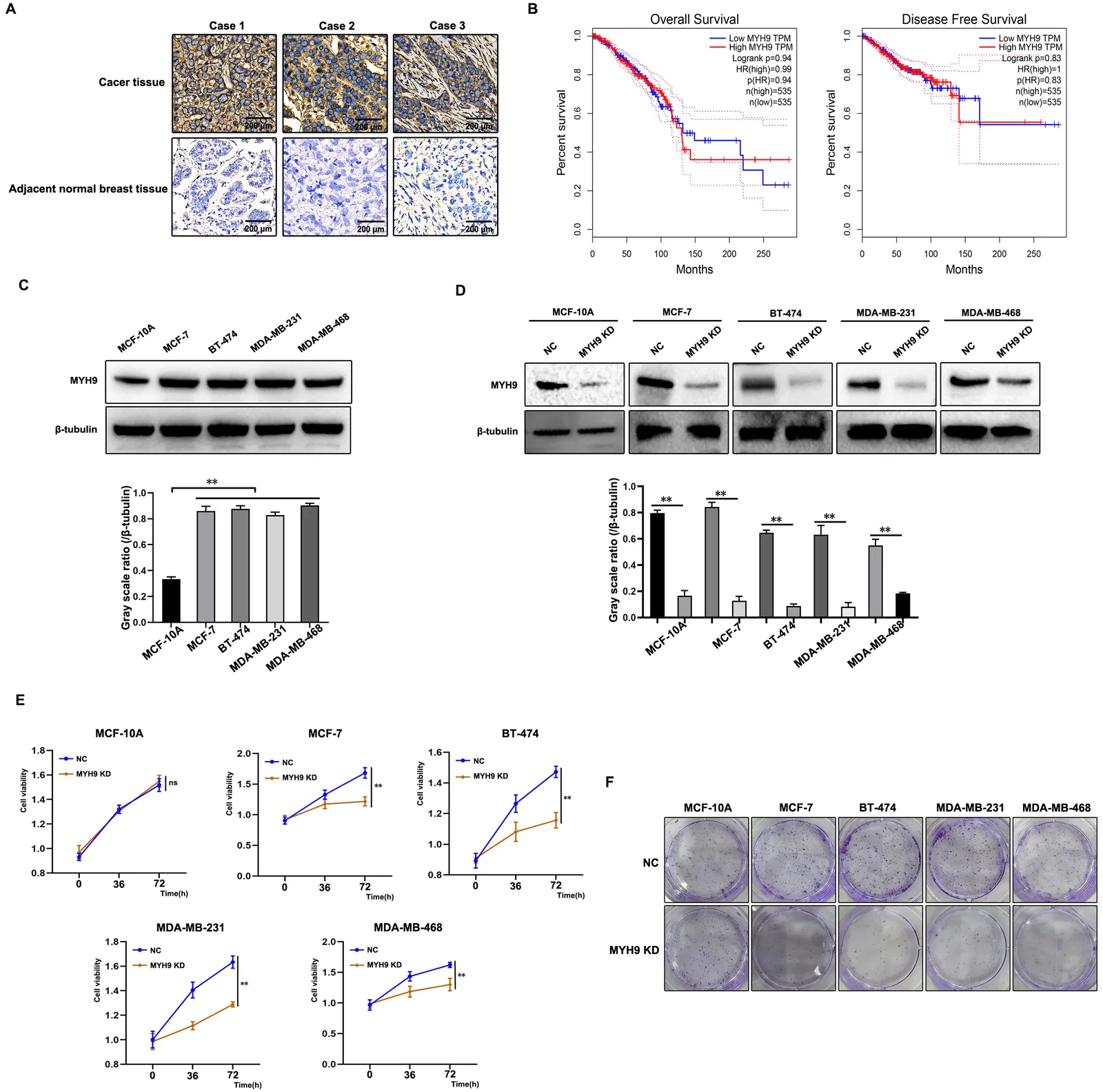
MYH9 exhibits high expression in breast cancer and promotes the proliferation of breast cancer cells *in vitro*. **(A)** Immunohistochemical analysis of MYH9 protein in breast cancer tissue and adjacent normal breast tissue, with MYH9 protein detected using a MYH9-specific antibody (brown) and nuclei stained with hematoxylin (blue). **(B)** Kaplan-Meier survival curves from the GEPIA database showing no significant association between MYH9 expression and Overall Survival or Disease-Free Survival in the total breast cancer cohort. **(C)** MYH9 expression levels in various breast cancer cell lines (MCF-10A, MCF-7, BT-474, MDA-MB-231, and MDA-MB-468), evaluated by western blotting with β-tubulin as a control. Quantification of the expression level in a histogram is shown below. **(D)** Knock-down of MYH9 in various breast cancer cell lines, assessed by western blotting with β-tubulin as a control. Quantification of the expression level in a histogram is shown below. **(E)** Effect of MYH9 on cell viability, measured by MTS at different time points (0, 36, 72 h) with 1×10^4^ cells seeded into 96-well plates. Experiments were performed in triplicate (**P ≤ 0.01). **(F)** Colony formation assay assessing the effect of MYH9 on cell proliferation, with 500 cells per well seeded in six-well plates and cultured for 15 days. NC means negative control; KD means knock down. “ns” stands for “not significant” (P > 0.05).

**Table 1:** MYH9 exhibits elevated expression in breast cancer tissues.

	N=175	MYH9		χ^2^	P
		High(n=128)	Low(n=47)		
Tissue				16.378	<0.001
Carcinoma tissue	145	n=115	n=30		
Para-carcinoma tissue	30	n=13	n=17		

**Table 2:** MYH9 expression levels showed a positive correlation with both tumor stage and lymph node metastasis status.

	N=145	MYH9		χ^2^	P
		High(n=115)	Low(n=30)		
Group					
Age at diagnosis				2.325	0.12731
<55	94	71	23		
≥55	51	44	7		
Stage				12.233	<0.01
I	30	18	12		
II	70	55	15		
III	45	42	3		
Lymph node metastasis				4.959	<0.05
Positive	49	44	5		
Negative	96	71	25		

**Table 3:** Association between MYH9 expression and molecular subtypes in breast cancer.

Molecular subtype	n	MYH9 high [n (%)]	MYH9 low [n (%)]	P value
Triple-negative (TNBC)	42	40 (95.2%)	2 (4.8%)	<0.001
Luminal A	13	8 (61.5%)	5 (38.5%)	0.1701
Luminal B	34	22 (64.7%)	12 (35.3%)	0.0501
HER2-enriched	20	14 (70.0%)	6 (30.0%)	0.3929

To investigate the expression of MYH9 in breast cancer, we utilized one normal breast epithelial cell line (MCF-10A) and four breast cancer cell lines (MCF-7, BT-474, MDA-MB-231, and MDA-MB-468). Western blot analysis revealed a marked upregulation of MYH9 expression in breast cancer cells compared to normal breast epithelial cells (**Figure 1C**). Following stable knockdown of MYH9 in each cell line (**Figure 1D**), MTS (**Figure 1E**) and colony formation assays (**Figure 1F**) indicated that MYH9 depletion significantly suppressed the proliferative capacity of breast cancer cells *in vitro*, while exerting no significant effect on the proliferation of normal breast epithelial cells.

Collectively, our experimental data indicate that MYH9 exhibits elevated expression in breast cancer tissues and cell lines, and is associated with tumor proliferation and disease progression.

### 3.2 MYH9 promotes ADR resistance of breast cells *in vitro* and *in vivo*

We selected the MCF-7 and MDA-MB-231 cell lines to conduct a more in-depth investigation into the impact of MYH9 on ADR resistance in breast cancer cells. The results of the MTS assay demonstrated that the proliferative capacity of MCF-7 and MDA-MB-231 cells gradually declined under ADR treatment (**Figure 2A**). Simultaneously, the Western blot results revealed that the expression of MYH9 in both cell lines gradually augmented with ADR treatment (**Figure 2B**). To explore the impact of MYH9 on the drug resistance of breast cancer cells, we overexpressed MYH9 in MCF-7 and MDA-MB-231 cells (**Figure 2C**) and intervened in their growth using ADR. The MTS assay results demonstrated that the overexpression of MYH9 notably enhanced the proliferation ability of cells in the ADR environment when compared with the control group (**Figure 2D**). Subsequently, we established ADR-resistant models of MCF-7 and MDA-MB-231 cells by means of the increasing concentration method of ADR. The MTS assay results revealed that the resistant cells were capable of stable proliferation in the ADR environment (**Figure 2E**). IC50 curves demonstrated that the ADR-resistant cell lines exhibited significantly reduced sensitivity to doxorubicin compared with their parental cells (**Figure 2F**). Furthermore, Western blot analysis showed that the expression of MYH9 and multidrug resistance-related proteins (P-gp, MRP1, and BCRP1) in the resistant cell lines was significantly up-regulated in comparison with the wild-type cells (**Figure 2G**). Knocking down MYH9 was confirmed by western blot (**Figure 2H**). MYH9 knockdown significantly inhibited the *in vitro* proliferation of ADR-resistant breast cancer cells (**Figure 2I**) and induced apoptosis as measured by Caspase-3/7 activity assay (**Figure 2J**). To investigate the effect of MYH9 on the *in vivo* drug resistance of breast cancer cells, we injected breast cancer resistant cell lines with or without MYH9 knockdown into the subcutaneous of nude mice and treated them with ADR. The tumor formation results indicated that knocking down MYH9 could significantly inhibit the *in vivo* tumor formation of resistant cells under ADR treatment (**Figure 2K**). The TUNEL assay showed that the apoptosis level in the tumor tissues of the MYH9 knockdown group was significantly elevated (**Figure 2L**). The above results imply that MYH9 can promote the *in vitro* and *in vivo* ADR resistance of breast cancer cells.

**Figure 2:**
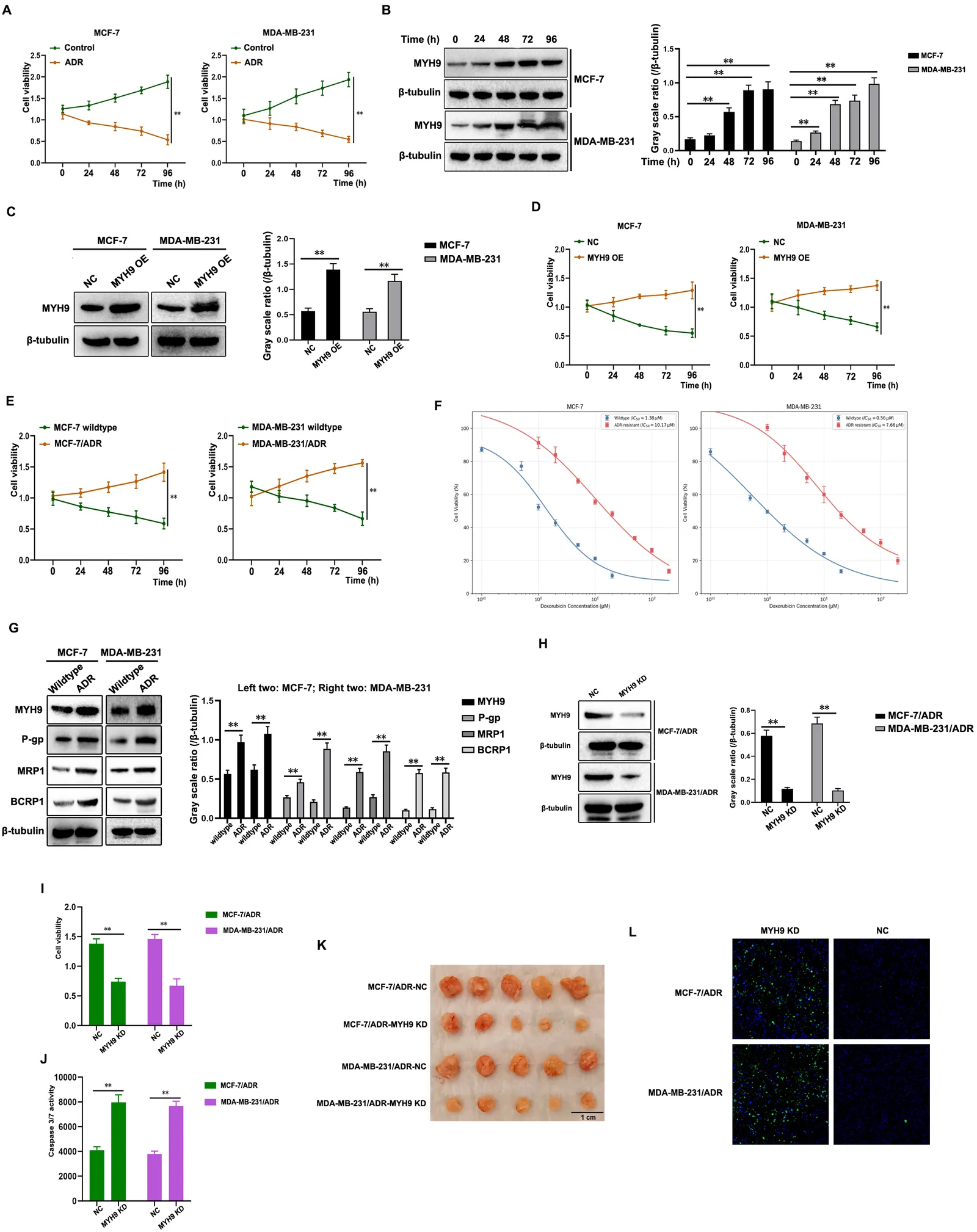
MYH9 promotes ADR resistance of breast cells *in vitro* and *in vivo*. **(A)** Effect of ADR on the viability of breast cancer cells. 1×10^4^ cells were seeded into 96-well plates. At different time points after treatment with 200 nM ADR cell viability was measured by MTS. The experiments were carried out in triplicate (**P ≤ 0.01). **(B)** Effect of ADR on the expression of MYH9 at different time points, followed by Western blot analysis of MYH9 level. β-tubulin was used as internal control. Quantification of the expression level in a histogram is shown right. **(C)** Overexpression of MYH9 in MDA-MB-231 and MCF-7 cells, assessed by western blotting with β-tubulin as a control. Quantification of the expression level in a histogram is shown right. **(D)** MTS assay to assess the impact of MYH9 overexpression on cellular proliferation at a concentration of 1 μM ADR. **(E)** Viability of MCF-7/ADR and MDA-MB-231/ADR cells treated with 1 μM ADR. MTS was used to measure cell viabilities at different time points. The experiments were carried out in triplicate (**P ≤ 0.01). **(F)** IC50 curves showing the drug sensitivity of parental and ADR-resistant cell lines to doxorubicin. **(G)** Western blot analysis of MYH9 and multidrug resistance-related proteins (P-gp, MRP1, BCRP1) in wild-type and ADR-resistant cell lines. β-tubulin was used as internal control. Quantification of the expression level in a histogram is shown right. **(H)** Knock down of MYH9 in MDA-MB-231/ADR and MCF-7/ADR cells, assessed by western blotting with β-tubulin as a control. Quantification of the expression level in a histogram is shown right. **(I)** MTS assay to assess the impact of MYH9 knockdown on cellular proliferation at a concentration of 1 μM ADR (**P ≤ 0.01). **(J)** The role of MYH9 in the regulation of ADR-resistant cell apoptosis. Apoptosis was examined with the Caspase-3/7 activity assay (**P ≤ 0.01). **(K)** The effect of MYH9 expression on tumorigenesis of ADR-resistant cells *in vivo*. Mice received a subcutaneous injection of 200 μL (1 x 10^5^ cells) of the cell suspension to induce tumor formation. Seven days later, the mice were intraperitoneally injected with ADR (30 mg/kg) every three days for 4 weeks. **(L)** The role of MYH9 in the regulation of ADR-resistant cell apoptosis *in vivo*. Apoptosis was examined with the TUNEL assay. NC means negative control; KD means knock down; OE means overexpression.

### 3.3 MYH9 inhibit apoptosis through the ERS pathway in breast cancer cells

The ERS response is frequently linked to the development of drug resistance. Our findings demonstrate that treatment of ADR-resistant breast cancer cells with an ER stress inducer (TG) markedly enhances their sensitivity to ADR and promotes apoptosis (**Figures 3A, B**). In contrast, administration of an ER stress inhibitor (4-PBA) to wild-type breast cancer cells significantly diminishes chemosensitivity and reduces apoptotic levels (**Figures 3A, B**). Western blot analysis reveals a substantial upregulation of the key ER stress marker GRP78 in drug-resistant breast cancer cell lines, accompanied by suppression of downstream PERK pathway-mediated apoptotic protein expression (**Figure 3C**). These results imply that the ER stress-mediated apoptotic pathway is functionally suppressed during the acquisition of drug resistance in breast cancer. We further investigated the impact of MYH9 expression on ERS response in breast cancer cells. Western blot analysis revealed that knockdown of MYH9 in drug-resistant breast cancer cells markedly reduced the expression of the key ER stress marker GRP78, while increasing PERK phosphorylation and upregulating pro-apoptotic proteins Bax and Bad, alongside downregulation of the anti-apoptotic protein Bcl-2 (**Figure 3D**). Furthermore, we evaluated the downstream components of the PERK signaling pathway to confirm its activation. As shown in **Figure 3D**, knockdown of MYH9 significantly increased the phosphorylation levels of eIF2α (p-eIF2α) and upregulated the expression of downstream transcription factors ATF4 and CHOP in ADR-resistant cells. To verify the specificity of the MYH9-GRP78 interaction, co-immunoprecipitation assays demonstrated that MYH9 specifically binds to GRP78 but not to other ER chaperones such as CALR or GRP94 (**Figure 3E**). The effect of MYH9 silencing on ER stress-related protein expression was further assessed in animal models. Immunohistochemical data demonstrated that MYH9 knockdown suppressed GRP78 expression *in vivo* and enhanced PERK phosphorylation, while also reducing the expression of the proliferation marker Ki67 (**Figure 3F**). These findings suggest that MYH9 promotes ADR resistance in breast cancer cells by suppressing ER stress-mediated apoptosis both *in vitro* and *in vivo*.

**Figure 3:**
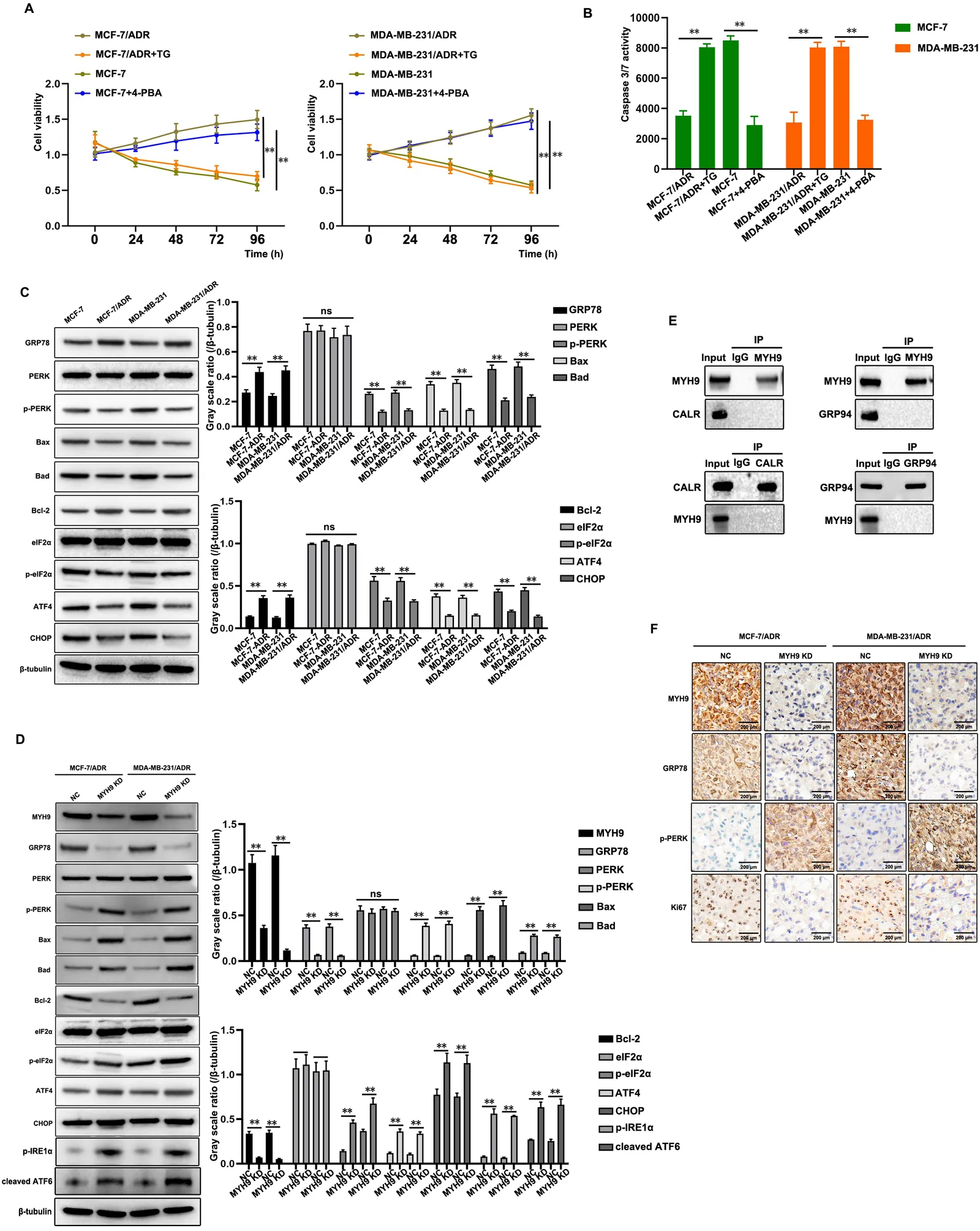
MYH9 inhibits apoptosis through the ERS pathway in breast cancer cells. **(A)** The influence of ERS response on ADR resistance in breast cancer cells. The ERS was inhibited or induced in cells by using ERS inhibitor (4-PBA) or inducer (TG) respectively. Cellular proliferation was examined with the MTS assay. **(B)** Apoptosis was examined with the Caspase-3/7 activity assay (**P ≤ 0.01). **(C)** The levels of ERS related proteins and downstream apoptotic pathway related proteins were analyzed using Western blot in ADR-resistant cells. Quantification of the expression level in a histogram is shown right. **(D)** Western blot analysis was used to assess the impact of MYH9 knockdown on the expression of ERS related proteins and downstream apoptotic pathway related proteins in ADR-resistant cells. Quantification of the expression level in a histogram is shown right. **(E)** Co-immunoprecipitation assays demonstrating the specificity of MYH9 interaction with GRP78, showing no interaction with other ER chaperones (CALR and GRP94). **(F)** Immunohistochemistry was used to assess the impact of MYH9 knockdown on the expression of ERS related proteins in animal models. NC means negative control; KD means knock down. “ns” stands for “not significant” (P > 0.05).

### 3.4 MYH9 interacts with GRP78 and inhibits its ubiquitination and degradation

Our investigation revealed that acquired ADR resistance in breast cancer cells correlates with MYH9 expression and ERS. We further established that MYH9 sustains high expression levels of the key ER stress chaperone GRP78. Subsequent analysis demonstrated a time-dependent upregulation of both MYH9 and GRP78 protein expression upon ADR treatment (**Figure 4A**). To investigate whether MYH9 affects GRP78 protein stability, we performed CHX chase assays. Knockdown of MYH9 in drug-resistant cells resulted in accelerated degradation of GRP78 (**Figure 4B**), whereas overexpression of MYH9 stabilized GRP78 protein levels (**Figure 4C**). We further hypothesized that MYH9 regulates GRP78 stability through proteasomal degradation. This was confirmed by treating cells with the proteasome inhibitor MG-132, which markedly attenuated the GRP78 degradation induced by MYH9 depletion (**Figure 4D**). Co-immunoprecipitation assays verified a direct physical interaction between MYH9 and GRP78 in ADR-resistant cell lines (**Figure 4E**). The interaction between exogenous GRP78 and MYH9 was further demonstrated in HEK293T cells (**Figure 4F**). Moreover, MYH9 knockdown significantly enhanced the ubiquitination of GRP78 in resistant cells (**Figure 4G**), whereas MYH9 overexpression suppressed GRP78 ubiquitination (**Figure 4H**). Collectively, these data indicate that in drug-resistant breast cancer cells, MYH9 directly interacts with GRP78 to inhibit its ubiquitin-mediated proteasomal degradation.

**Figure 4:**
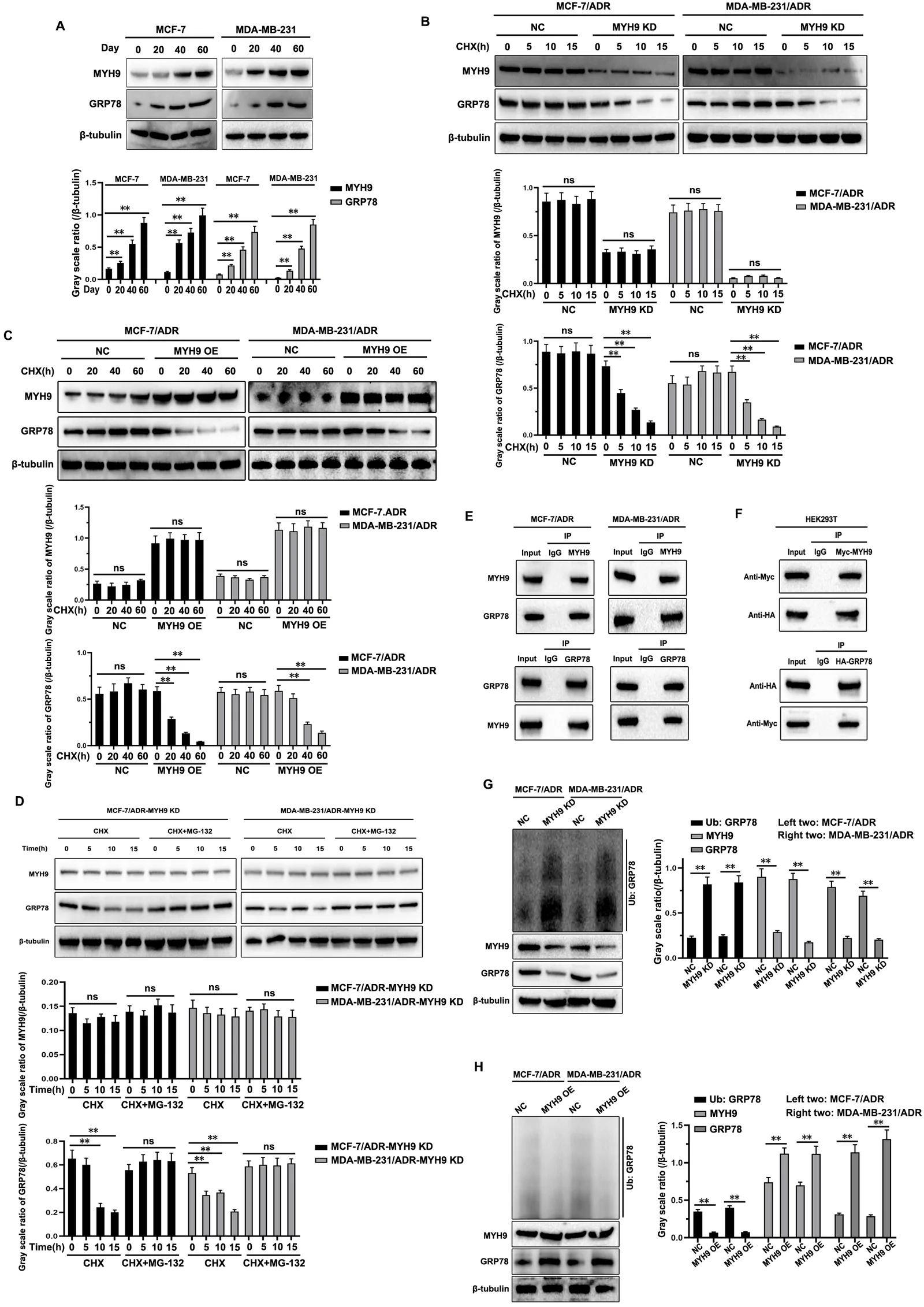
MYH9 interacts with GRP78 and inhibits its ubiquitination and degradation. **(A)** Western blot analysis showing the time-dependent upregulation of MYH9 and GRP78 protein expression upon ADR treatment in MCF-7 and MDA-MB-231 cells. Quantification of the expression level in a histogram is shown below. **(B)** GRP78 protein stability in the presence of CHX after MYH9 knockdown assessed by Western blot with β-tubulin as a control. Relative protein expression level is shown below. Quantification of the expression level in a histogram is shown below. **(C)** GRP78 protein stability in the presence of CHX in MYH9 overexpression cells assessed by Western blot with β-tubulin as a control. Relative protein expression level is shown below. Quantification of the expression level in a histogram is shown below. **(D)** GRP78 protein stability in the presence of CHX and MG132 in MYH9 knockdown cells assessed by Western blot with β-tubulin as a control. Relative protein expression level is shown below. Quantification of the expression level in a histogram is shown below. **(E)** Co-IP assays demonstrating MYH9 binding to GRP78 in ADR-resistant cell lines. **(F)** The interaction of GRP78 and MYH9 in HEK293T cells transfected with HA-GRP78 and Myc-MYH9. **(G)** GRP78 ubiquitination after MYH9 knockdown in ADR-resistant cell lines, using Co-IP with an anti-GRP78 antibody and immunoblotting (IB) with an anti-ubiquitin (Ub) antibody. Quantification of the expression level in a histogram is shown right. **(H)** GRP78 ubiquitination after MYH9 overexpression in ADR-resistant cell lines, using Co-IP with an anti-GRP78 antibody and immunoblotting (IB) with an anti-ubiquitin (Ub) antibody. Quantification of the expression level in a histogram is shown right. NC means negative control; KD means knock down; OE means overexpression. “ns” stands for “not significant” (P > 0.05).

### 3.5 MYH9 promotes ADR resistance in breast cancer by inhibiting the ERS-mediated apoptotic pathway through GRP78

To elucidate the role of MYH9 in modulating drug resistance in breast cancer cells via inhibition of GRP78 protein degradation, we performed MYH9 knockdown combined with GRP78 overexpression in drug-resistant breast cancer cells and assessed alterations in cellular sensitivity to ADR. MTS assays revealed that MYH9 depletion significantly decreased cell viability at 36 and 72 hours, whereas concurrent MYH9 knockdown and GRP78 overexpression partially restored proliferative capacity to baseline levels (**Figure 5A**). Consistently, Caspase 3/7 activity assays demonstrated that MYH9 silencing significantly promoted apoptosis *in vitro*, and this pro-apoptotic effect was effectively reversed upon GRP78 overexpression (**Figure 5B**). Western blot analysis further demonstrated that MYH9 knockdown not only reduced MYH9 and GRP78 protein levels but also triggered the phosphorylation of PERK and its downstream effectors, including p-eIF2α, ATF4, and CHOP. Furthermore, MYH9 depletion shifted the balance of apoptosis-related proteins by upregulating pro-apoptotic factors (Bax and Bad) and downregulating the anti-apoptotic factor Bcl-2. Strikingly, when GRP78 was overexpressed alongside MYH9 knockdown, the GRP78-mediated ERS-associated apoptotic signaling was effectively suppressed, as evidenced by the restoration of GRP78 levels, the reduction of p-PERK, p-eIF2α, ATF4, and CHOP, and the recovery of Bcl-2 expression (**Figure 5C**).

**Figure 5:**
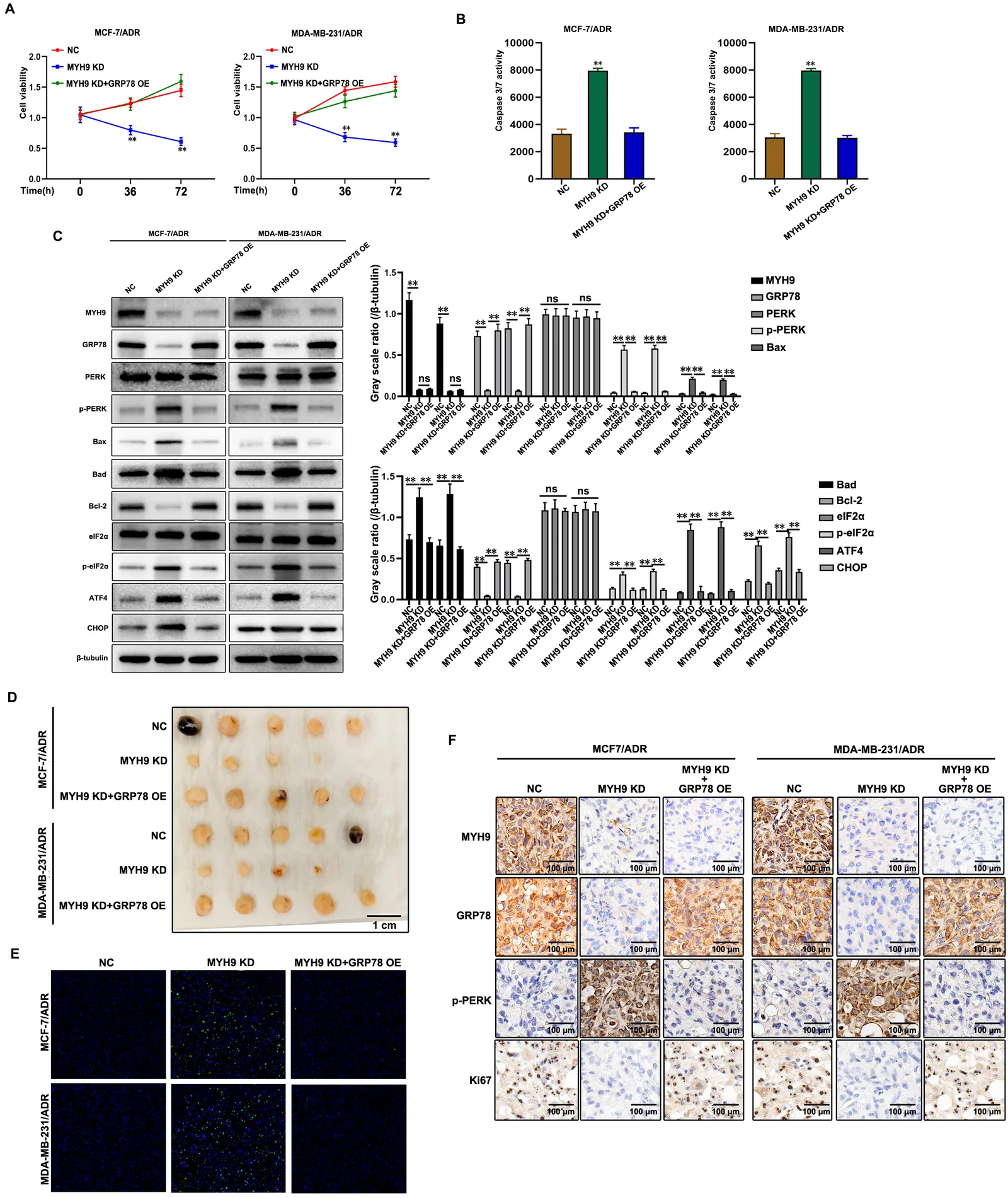
MYH9 promotes ADR resistance by inhibiting the ERS-mediated apoptotic pathway through GRP78. **(A)** MTS assay assessing the influence of the MYH9/GRP78 axis on the proliferation of ADR-resistant MCF-7/ADR and MDA-MB-231/ADR cells at 0, 36, and 72 hours. NC, negative control shRNA; KD, MYH9 knockdown; OE, GRP78 overexpression (**P ≤ 0.01). **(B)** Caspase-3/7 activity assay assessing the influence of the MYH9/GRP78 axis on the apoptosis of ADR-resistant cells (**, *P ≤* 0.01). **(C)** Western blot analysis evaluating the expression levels of ERS-related proteins and downstream apoptotic pathway proteins. β-tubulin was used as the loading control. Quantification of the expression level in a histogram is shown right. **(D)** *In vivo* tumorigenesis assay of ADR-resistant cells. Mice received a subcutaneous injection of 200 μL (1 × 10^5^ cells) of the cell suspension to induce tumor formation. Seven days later, the mice were intraperitoneally injected with ADR (30 mg/kg) every three days for 4 weeks. Tumor photographs are shown. **(E)** The role of the MYH9/GRP78 axis in regulating ADR-resistant cell apoptosis *in vivo*. Apoptosis was examined with the TUNEL assay, green fluorescence indicates apoptotic cells. **(F)** The role of the MYH9/GRP78 axis in regulating the expression of ERS-related proteins and proliferation markers in animal models. Protein levels of MYH9, GRP78, p-PERK, and Ki67 were examined with immunohistochemistry, brown staining indicates positive expression. NC means negative control; KD means knock down; OE means overexpression. “ns” stands for “not significant” (P > 0.05).

Our *in vivo* studies demonstrated that MYH9 knockdown significantly attenuated the tumorigenic capacity of breast cancer cells following ADR treatment, resulting in markedly smaller tumor volumes compared to the control group (**Figure 5D**). However, concurrent suppression of MYH9 and overexpression of GRP78 restored tumor formation ability to levels comparable with control groups (**Figure 5D**). TUNEL assays further indicated that MYH9 inhibition increased the number of apoptotic cells *in vivo*, whereas GRP78 overexpression significantly reduced this apoptosis (**Figure 5E**). Immunohistochemical staining confirmed that MYH9 knockdown downregulated GRP78 expression, increased PERK phosphorylation, and decreased Ki67-positive proliferative cells in tumor tissues; notably, these molecular and phenotypic changes were reverted by GRP78 overexpression (**Figure 5F**). Collectively, these results suggest that elevated MYH9 expression in breast cancer cells impedes GRP78 ubiquitination and degradation via direct interaction, leading to suppression of downstream PERK phosphorylation and apoptosis signaling, thereby fostering chemoresistance.

## 4 Discussion

The role of MYH9 in malignant tumors has garnered increasing interest in recent years. Current evidence indicates that MYH9 is highly expressed in a range of cancer cell types, including hepatocellular carcinoma, lung cancer, gastric cancer, and esophageal carcinoma, where it correlates with tumor progression and poor prognosis (11–20). While prior studies have established the significance of MYH9 in tumor biology, its function in breast cancer—particularly concerning drug resistance regulation—remains poorly understood. In this study, we observed elevated MYH9 expression in breast cancer tissues and cell lines, which correlated with disease stage and lymph node metastasis. Through *in vitro* experiments, we demonstrated that knockdown of MYH9 significantly suppresses breast cancer cell proliferation, indicating that MYH9 exerts a pro-tumorigenic function in breast cancer.

Building on these findings, we further examined the clinical implications of MYH9 expression. Survival analysis based on the GEPIA database did not reveal a significant correlation between MYH9 and overall survival in the general breast cancer population, a result likely attributable to the high molecular heterogeneity of this disease. Notably, however, our data demonstrated that MYH9 is specifically upregulated in the triple-negative breast cancer (TNBC) subtype, whereas its expression varies across other subtypes. This subtype-specific upregulation is clinically significant: since TNBC lacks targeted therapies and relies heavily on chemotherapy, the role of MYH9 in driving malignant phenotypes and conferring drug resistance—as evidenced by our functional assays—suggests that MYH9 could serve as a potential therapeutic target specifically for TNBC patients, rather than a general prognostic marker for all breast cancers.

Given TNBC’s heavy reliance on chemotherapy, understanding the mechanisms of chemoresistance is particularly urgent. The primary or acquired resistance of tumor cells to chemotherapeutic agents represents a fundamental mechanism underlying treatment failure, primarily characterized by evasion of drug-induced apoptosis. ADR, an anthracycline antibiotic widely used in the treatment of breast cancer and other malignancies, exerts its antitumor effects through disruption of DNA replication and transcription and induction of apoptotic cell death (32). However, drug resistance develops in approximately 30-50% of patients, ultimately leading to disease recurrence (32, 33). Here, we report that ADR upregulates MYH9 expression while suppressing breast cancer cell proliferation. MYH9 overexpression significantly diminished cellular sensitivity to ADR, and in established ADR-resistant cell lines, MYH9 expression was elevated; its knockdown markedly suppressed cell proliferation *in vitro* and tumor growth *in vivo* under ADR treatment, while enhancing apoptosis. Collectively, these results establish MYH9 as a key promoter of ADR resistance in breast cancer.

To elucidate the mechanism underlying MYH9-mediated ADR resistance, we investigated the ERS pathway. ERS is triggered by the accumulation of misfolded proteins under conditions such as hypoxia, chemotherapeutic treatment, or oxidative stress, activating the UPR mediated by three principal sensors: PERK, ATF6, and IRE1 (23, 24). While ATF6 and IRE1 signaling primarily augment the clearance of misfolded proteins, the PERK pathway promotes apoptosis through activation of downstream effectors (28, 29). Under physiological conditions, these sensors remain inactive through association with the chaperone GRP78; upon stress, misfolded proteins compete for GRP78 binding, initiating protective signaling (28, 29). ERS exhibits a dual function in chemotherapy resistance: initially promoting apoptosis, but prolonged treatment induces adaptive responses that suppress apoptosis and confer drug resistance (25–28). In this study, modulation of the ERS response correspondingly altered ADR sensitivity. Suppression of ER stress upregulated GRP78 while attenuating PERK-mediated apoptotic signaling. Notably, knockdown of MYH9 in resistant cells substantially reduced GRP78 expression and increased phosphorylation of PERK and its downstream targets, including p-eIF2α, ATF4, and CHOP, while also activating the IRE1 and ATF6 branches of the UPR. These findings indicate that MYH9 promotes ADR resistance by inactivating the ER stress-induced apoptotic pathway across multiple UPR branches.

Having established the functional role of MYH9 in regulating ERS, we next investigated the molecular mechanism. Previous studies have indicated that cytoplasmic MYH9 can directly interact with tumor-associated functional proteins, modulating the stability of target proteins (14–18). In this study, we observed that during ADR-induced drug resistance, MYH9 and GRP78 expression increased progressively with a significant positive correlation. Knockdown of MYH9 reduced the half-life and stability of GRP78 protein. Co-immunoprecipitation and ubiquitination assays revealed that MYH9 directly binds to GRP78, inhibiting its ubiquitination and subsequent degradation. Importantly, this interaction is highly specific, as MYH9 did not bind to other ER chaperones such as CALR or GRP94. *In vitro* and *in vivo* experiments confirmed that MYH9 knockdown suppressed proliferation and promoted apoptosis under ADR treatment, whereas co-expression of GRP78 restored these phenotypes to baseline levels. Thus, MYH9 stabilizes GRP78 to attenuate PERK phosphorylation and downstream apoptotic signaling, thereby enhancing drug resistance.

Several limitations of this study warrant consideration. First, our clinical cohort was retrospective and relatively small, lacking detailed information on specific chemotherapy regimens and long-term follow-up. Second, our mechanistic insights were primarily derived from cell line models and xenografts, which may not fully recapitulate the complex tumor microenvironment. Future studies should validate these findings in larger, prospective clinical cohorts and explore whether targeting the MYH9-GRP78 axis can effectively sensitize TNBC tumors to ADR-based therapies.

## Data Availability

The raw data supporting the conclusions of this article will be made available by the authors, without undue reservation.

## References

[1] Xiong X Zheng LW Ding Y Chen YF Cai YW Wang LP . Breast cancer: pathogenesis and treatments. Signal Transduct Target Ther. (2025) 10:49. doi: 10.1038/s41392-024-02108-4 39966355 PMC11836418

[2] Yan Q Deng Y Zhang Q . A comprehensive overview of metaplastic breast cancer: Features and treatments. Cancer Sci. (2024) 115:2506–14. doi: 10.1111/cas.16208 38735837 PMC11309924

[3] Khan MM Yalamarty SSK Rajmalani BA Filipczak N Torchilin VP . Recent strategies to overcome breast cancer resistance. Crit Rev Oncol Hematol. (2024) 197:104351. doi: 10.1016/j.critrevonc.2024.104351 38615873

[4] Kerr AJ Dodwell D McGale P Holt F Duane F Mannu G . Adjuvant and neoadjuvant breast cancer treatments: A systematic review of their effects on mortality. Cancer Treat Rev. (2025) 105:102375. doi: 10.1016/j.ctrv.2022.102375 PMC9096622 35367784

[5] Wu GY Xiao MZ Hao WC Yang ZS Liu XR Xu DS . Drug resistance in breast cancer: Mechanisms and strategies for management. Drug Resist Update. (2025) 83:101288. doi: 10.1016/j.drup.2025.101288 40795794

[6] Yamazaki R Ohno N . Myosin superfamily members during myelin formation and regeneration. J Neurochem. (2024) 168:2264–74. doi: 10.1111/jnc.16202 39136255

[7] Hartman MA Spudich JA . The myosin superfamily at a glance. J Cell Sci. (2012) 125:1627–32. doi: 10.1242/jcs.094300 22566666 PMC3346823

[8] Sweeney HL Houdusse A Robert-Paganin J . Myosin structures. Adv Exp Med Biol. (2020) 1239:7–19. doi: 10.1007/978-3-030-38062-5_2 32451853

[9] Li YR Yang WX . Myosin superfamily: The multi-functional and irreplaceable factors in spermatogenesis and testicular tumors. Gene. (2016) 576:195–207. doi: 10.1016/j.gene.2015.10.022 26478466

[10] Pecci A Ma X Savoia A Adelstein RS . MYH9: Structure, functions and role of non-muscle myosin IIA in human disease. Gene. (2018) 664:152–67. doi: 10.1016/j.gene.2018.04.048 29679756 PMC5970098

[11] Liu Q Cheng C Huang J Yan W Wen Y Liu Z . MYH9: A key protein involved in tumor progression and virus-related diseases. BioMed Pharmacother. (2024) 171:116118. doi: 10.1016/j.biopha.2023.116118 38181716

[12] Li Q Luo H Dai FQ Wang RT Fan XQ Luo YY . SAMD9 promotes postoperative recurrence of esophageal squamous cell carcinoma by stimulating MYH9-mediated GSK3β/β-catenin signaling. Adv Sci (Weinh). (2023) 10:e2203573. doi: 10.1002/advs.202203573 PMC10104667 36757050

[13] Shi Y Zhao Z Zhou S Zhou Z Huang Z Zhou Z . CHD4 drives gastric cancer metastasis via MYH9/GSK3β/β-catenin axis and WNT/EMT pathway activation. Cancer Lett. (2025) 628:217813. doi: 10.1016/j.canlet.2025.217813 40480417

[14] Hu R Cao Y Wang Y Zhao T Yang K Fan M . TMEM120B strengthens breast cancer cell stemness and accelerates chemotherapy resistance via β1-integrin/FAK-TAZ-mTOR signaling axis by binding to MYH9. Breast Cancer Res. (2024) 26:48. doi: 10.1186/s13058-024-01802-z 38504374 PMC10949598

[15] Ye G Yang Q Lei X Zhu X Li F He J . Nuclear MYH9-induced CTNNB1 transcription, targeted by staurosporin, promotes gastric cancer cell anoikis resistance and metastasis. Theranostics. (2020) 10:7545–60. doi: 10.7150/thno.46001 32685004 PMC7359096

[16] Shan Q Yin L Zhan Q Yu J Pan S Zhuo J . The p-MYH9/USP22/HIF-1α axis promotes lenvatinib resistance and cancer stemness in hepatocellular carcinoma. Signal Transduct Target Ther. (2024) 9:249. doi: 10.1038/s41392-024-01963-5 39300073 PMC11412978

[17] Chen M Sun LX Yu L Liu J Sun LC Yang ZH . MYH9 is crucial for stem cell-like properties in non-small cell lung cancer by activating mTOR signaling. Cell Death Discov. (2021) 7:282. doi: 10.1038/s41420-021-00681-z 34635641 PMC8505404

[18] Yang JS Cao JM Sun R Zhou XJ Chen ZH Liu BW . SMYD4 promotes MYH9 ubiquitination through lysine monomethylation modification to inhibit breast cancer progression. Breast Cancer Res. (2025) 27:20. doi: 10.1186/s13058-025-01973-3 39930544 PMC11812198

[19] Song JX Wang Y Hua ZP Huang Y Hu LF Tian MR . FATS inhibits the Wnt pathway and induces apoptosis through degradation of MYH9 and enhances sensitivity to paclitaxel in breast cancer. Cell Death Dis. (2024) 15:835. doi: 10.1038/s41419-024-07164-w 39550407 PMC11569202

[20] Garlapati C Joshi S Yang C Chandrashekar DS Rida P Aneja R . A novel role for KIFC1-MYH9 interaction in triple-negative breast cancer aggressiveness and racial disparity. Cell Commun Signal. (2024) 22:312. doi: 10.1186/s12964-024-01664-0 38902769 PMC11188183

[21] Oakes SA Papa FR . The role of endoplasmic reticulum stress in human pathology. Annu Rev Pathol. (2015) 10:173–94. doi: 10.1146/annurev-pathol-012513-104649 25387057 PMC5568783

[22] Matsushima G Yanase Y Nakagawa T Goda M Ozawa K Hosoi T . Endoplasmic reticulum stress and unfolded protein response in immune cell function. Front Immunol. (2025) 16:1694102. doi: 10.3389/fimmu.2025.1694102 41208954 PMC12592176

[23] Hetz C . The unfolded protein response: controlling cell fate decisions under ER stress and beyond. Nat Rev Mol Cell Biol. (2012) 13:89–102. doi: 10.1038/nrm3270 22251901

[24] Wang M Law ME Castellano RK Law BK . The unfolded protein response as a target for anticancer therapeutics. Crit Rev Oncol Hematol. (2018) 127:66–79. doi: 10.1016/j.critrevonc.2018.05.003 29891114

[25] Khaled J Kopsida M Lennernäs H Heindryckx F . Drug resistance and endoplasmic reticulum stress in hepatocellular carcinoma. Cells. (2022) 11:632. doi: 10.3390/cells11040632 35203283 PMC8870354

[26] Panda SK Sanchez-Pajares IR Rehman A Del Vecchio V Mele L Chipurupalli S . ER stress and/or ER-phagy in drug resistance? Three coincidences are proof. Cell Commun Signal. (2025) 23:223. doi: 10.1186/s12964-025-02232-w 40361118 PMC12070796

[27] Ibrahim IM Abdelmalek DH Elfiky AA . GRP78: A cell's response to stress. Life Sci. (2019) 226:156–63. doi: 10.1016/j.lfs.2019.04.022 30978349 PMC7094232

[28] Liu Z Liu G Ha DP Wang J Xiong M Lee AS . ER chaperone GRP78/BiP translocates to the nucleus under stress and acts as a transcriptional regulator. Proc Natl Acad Sci USA. (2023) 120:e2303448120. doi: 10.1073/pnas.2303448120 37487081 PMC10400976

[29] Rozpedek W Pytel D Mucha B Leszczynska H Diehl JA Majsterek I . The role of the PERK/eIF2α/ATF4/CHOP signaling pathway in tumor progression during endoplasmic reticulum stress. Curr Mol Med. (2016) 16:533–44. doi: 10.2174/1566524016666160523143937 27211800 PMC5008685

[30] Jin J Ma Y Tong X Yang W Dai Y Pan Y . Metformin inhibits testosterone-induced endoplasmic reticulum stress in ovarian granulosa cells via inactivation of p38 MAPK. Hum Reprod. (2020) 35:1145–58. doi: 10.1093/humrep/deaa180 32372097 PMC7259369

[31] Liu X Lv Z Zou J Liu X Ma J Wang J . Afatinib down-regulates MCL-1 expression through the PERK-eIF2α-ATF4 axis and leads to apoptosis in head and neck squamous cell carcinoma. Am J Cancer Res. (2016) 6:1708–19. PMC5004074 27648360

[32] Bisht A Avinash D Sahu KK Patel P Das Gupta G Kurmi BD . A comprehensive review on doxorubicin: mechanisms, toxicity, clinical trials, combination therapies and nanoformulations in breast cancer. Drug Delivery Transl Res. (2025) 15:102–33. doi: 10.1007/s13346-024-01648-0 38884850

[33] Rivankar S . An overview of doxorubicin formulations in cancer therapy. J Cancer Res Ther. (2014) 10:853–8. doi: 10.4103/0973-1482.139267 25579518

