# Supplementary material for: MYH9 confers doxorubicin resistance by stabilizing GRP78 and suppressing the PERK signaling-driven apoptotic pathway in breast cancer
Source: Front Oncol. 2026 Oct 2;16:1923603. doi: 10.3389/fonc.2026.1923603 (PMC13672191; doi:10.3389/fonc.2026.1923603)
Supplement: Supplementary file 1 [file Table1.docx]

**The antibodies used in this study**

| Number | Name | Brand | Code | Purpose |
| --- | --- | --- | --- | --- |
| 1 | MYH9 | Proteintech | 11128-1-AP | Immunohistochemistry, Western blot, Co-IP |
| 2 | β-tubulin | Proteintech | 10094-1-AP | Western blot |
| 3 | GRP78 | Proteintech | 11587-1-AP | Immunohistochemistry, Western blot, Co-IP |
| 4 | PERK | Proteintech | 24390-1-AP | Western blot |
| 5 | p-PERK | Proteintech | 29546-1-AP | Immunohistochemistry, Western blot |
| 6 | Bax | Proteintech | 50599-2-Ig | Western blot |
| 7 | Bad | Proteintech | 10435-1-AP | Western blot |
| 8 | Bcl-2 | Proteintech | 12789-1-AP | Western blot |
| 9 | Ki67 | Proteintech | 27309-1-AP | Immunohistochemistry |
| 10 | Ubiquitin | Proteintech | 10201-2-AP | Western blot |
| 11 | HA Tag | Proteintech | 51064-2-AP | Western blot |
| 12 | Myc Tag | Proteintech | 60003-2-Ig | Western blot |
| 13 | P-gp | Proteintech | 22336-1-AP | Western blot |
| 14 | MRP1 | Proteintech | 67228-1-Ig | Western blot |
| 15 | BCRP1 | Proteintech | 83835-1-RR | Western blot |
| 16 | p-eIF2α | Proteintech | 28740-1-AP | Western blot |
| 17 | ATF4 | Proteintech | 10835-1-AP | Western blot |
| 18 | CHOP | Proteintech | 15204-1-AP | Western blot |
| 19 | CALR | Proteintech | 27298-1-AP | Co-IP |
| 20 | GRP94 | Proteintech | 14700-1-AP | Co-IP |
